# The Application of Leaf Ultrasonic Resonance to *Vitis vinifera* L. Suggests the Existence of a Diurnal Osmotic Adjustment Subjected to Photosynthesis

**DOI:** 10.3389/fpls.2016.01601

**Published:** 2016-10-26

**Authors:** Domingo Sancho-Knapik, Hipólito Medrano, José J. Peguero-Pina, Maurizio Mencuccini, Maria D. Fariñas, Tomás G. Álvarez-Arenas, Eustaquio Gil-Pelegrín

**Affiliations:** ^1^Unidad de Recursos Forestales, Centro de Investigación y Tecnología Agroalimentaria de Aragón, Gobierno de AragónZaragoza, Spain; ^2^Centro de Investigación y Tecnología Agroalimentaria de Aragón, Instituto Agroalimentario de Aragón, Universidad de ZaragozaZaragoza, Spain; ^3^Research Group on Plant Biology under Mediterranean Conditions, Departament de Biologia, Universitat de les Illes BalearsPalma de Mallorca, Spain; ^4^ICREA at CREAFBarcelona, Spain; ^5^Sensors and Ultrasonic Technologies Department, Information and Physics Technologies Institute, Spanish National Research CouncilMadrid, Spain

**Keywords:** diurnal osmotic adjustment, drought, gas exchange, leaf ultrasonic frequency, *Vitis vinifera*, water potential

## Abstract

The main objective of this study was to apply the air-coupled broad-band ultrasonic spectroscopy in attached transpiring leaves of *Vitis vinifera* L. to monitor changes in leaf water potential (Ψ) through the measurements of the standardized value of the resonant frequency associated with the maximum transmitance (*f/f*_o_). With this purpose, the response of grapevine to a drought stress period was investigated in terms of leaf water status, ultrasounds, gas exchange and sugar accumulation. Two strong correlations were obtained between *f/f*_o_ and Ψ measured at predawn (pd) and at midday (md) with different slopes. This fact implied the existence of two values of Ψ for a given value of *f/f*_o_, which was taken as a sign that the ultrasonic technique was not directly related to the overall Ψ, but only to one of its components: the turgor pressure (*P*). The difference in Ψ at constant *f/f*_o_ (δ) was found to be dependent on net CO_2_ assimilation (*A*) and might be used as a rough estimator of photosynthetic activity. It was then, the other main component of Ψ, osmotic potential (π), the one that may have lowered the values of md Ψ with respect to pd Ψ by the accumulation of sugars associated to net CO_2_ assimilation. This phenomenon suggests the existence of a diurnal osmotic adjustment in this species associated to sugars production in well-watered plants.

## Introduction

Grapevines are one of the world’s most important commercial crops, not only from an economic point of view, but also because of the extension of its worldwide cultivation. A significant proportion of these vineyards grows under Mediterranean-type climates (Tonietto and Carbonneau, 2004), where *Vitis vinifera* L. has to cope with a summer water deficit that may affect berry quality (Chaves et al., 2007; Chaves et al., 2010). In such environments, climate change models (García-Mozo et al., 2010) predict an increment in extreme high temperature and drought events, which may represent a risk for the wine industry forcing it to a more frequent use of irrigation for affordable crop production (Chaves et al., 2007). The high water requirements of grapevines during the growing season (Netzer et al., 2005; Zhang et al., 2007) and the large dependence of berry quality parameters on soil water availability (Medrano et al., 2003; Keller et al., 2008; Flexas et al., 2010; Romero et al., 2010; Pou et al., 2011) justifies special attention in the optimization of water use during vineyard irrigation to achieve a more environmentally sustainable viticulture with a reasonable fruit quality (Jones, 2004).

Direct measurements of the plant stress response, ‘plant stress sensing’ according to Jones (2004), have been suggested as a better way to implement adequate irrigation scheduling compared to only estimating atmospheric water demand or soil moisture status (Jones, 1990, 2004, 2007). Among the methods that use the plant as an indicator, the air-coupled broad-band ultrasonic spectroscopy technique (Álvarez-Arenas et al., 2009; Sancho-Knapik et al., 2010, 2011, 2012), has been proven recently as a non-destructive, non-invasive and non-contact method for the dynamic determination of leaf water status in *V. vinifera* (Sancho-Knapik et al., 2013a; Fariñas et al., 2014). This method is based on the excitation of thickness resonances of the leaves and on the analysis of the spectral response in the vicinity of the first order thickness resonance (Álvarez-Arenas et al., 2009), and has been revealed as a good indicator of the water potential (Ψ) and the RWC of leaves through changes in the *f/f*_o_ at the maximum transmittance (Sancho-Knapik et al., 2010, 2013a). These changes in *f/f*_o_ that occur before turgor loss can be attributed to changes in the macroscopic effective elastic constant of the leaf in the thickness direction (*c*_33_) (Sancho-Knapik et al., 2011) that are mainly associated with changes in the tautness of the microfibrils in the cell wall (Gibson and Ashby, 1997) and therefore with changes in the bulk modulus of elasticity of the cell wall (𝜀 the change in turgor pressure for a given fractional change in the weight of symplasmic water) (Tyree, 1981; Sancho-Knapik et al., 2011). As 𝜀 is dependant on the variations in turgor pressure (Tyree and Jarvis, 1982), changes in *f/f*_o_ before the turgor loss point can be attributed mainly to the variations in turgor pressure.

Leaf Ψ is the sum of turgor pressure, or the outward hydrostatic pressure which is opposed by the cell wall (*P*, positive), osmotic potential, the component of the water potential due to the presence of solutes (π, negative) and matric potential, the component relative to the binding of water molecules to non-dissolves structures of the cells, which is negligible as compared with the other two (Koide et al., 1989). Loss of water from turgid attached leaf tissues in response to transpiration can decrease Ψ not only through a significant decline in *P*, but also through a decline in π. Although variations in π can occur following passive concentration of solutes, this is generally less important when compared with the variation in *P* (Richter, 1978; Koide et al., 1989), associating the decrease in Ψ when a turgid leaf losses water, mainly with a decrease in *P*. In this case, the relation between *f/f*_o_ and Ψ in attached leaves may be in line with previous works where processes occurring during leaf dehydration were studied (Sancho-Knapik et al., 2010, 2013a). On the other hand, photosynthetic products, besides being upload immediately to the phloem or converted into starch, can be accumulated, reducing also leaf π by a net increase in solutes (Acevedo et al., 1979). This process, called diurnal osmotic adjustment, has been revealed as a mechanism playing a key role in leaf expansion by minimizing the opportunities for significant water loss from leaf tissues (Acevedo et al., 1979; Girma and Krieg, 1992). Diurnal osmotic adjustment induces a significant reduction in Ψ without any decrease in *P*, so that, a variation in Ψ caused only by a net increase of solutes in the leaf may not induce a variation neither in RWC nor in *f/f*_o_. In this second case, the air-coupled broad-band ultrasonic spectroscopy technique may be used to appreciate mechanisms at leaf level that have been interpreted as the consequence of diurnal osmotic adjustment associated to photosynthesis when no changes in *f/f*_o_ occur during a decrease in Ψ. In this sense, the aim of this study is to apply the air-coupled broad-band ultrasonic spectroscopy on *V. vinifera* to (i) monitor changes in *f/f*_o_ with changes in leaf water potential on attached transpiring leaves and (ii) analyse the mechanisms that can suggest the existence of a diurnal osmotic adjustment associated with photosynthesis.

## Materials and Methods

### Plant Material and Experimental Conditions

Two-year-old rooted cuttings of *Vitis vinifera* cv. Grenache were planted in 75-L containers with a mixture of 80% compost (Neuhaus Humin Substrat N6; Klasman-Deilmann GmbH, Geeste, Germany) and 20% perlite. Plants grew during 2 years in a common garden located at CITA de Aragón (41°39′N, 0°52′W, Zaragoza, Spain; mean annual temperature 15.4°C, total annual precipitation 298 mm) with irrigation employed when necessary. Two weeks before the beginning of the experiment (starting date on 16 July 2014), three pots of each variety were placed under a transparent greenhouse tunnel of alveolar polycarbonate that allowed passing 90% of PPFD. The use of covers in water-stress experiments had the advantage of performing measurements in more controlled environmental conditions, avoiding re-watering by storms or unwanted rainfall events.

Watering was stopped on 15 July 2014 and measurements in well watered plants started on 16 July 2014. During the following days, measurements of water potential, gas exchange and ultrasounds were performed every 2 or 3 days with increasing levels of drought stress. pd measurements of ultrasounds and water potential (Ψ^pd^) were conducted strictly between 3 and 4 h (solar time), while md measurements of ultrasounds and water potential (Ψ^md^) were made at 12 h (solar time). Measurements of gas exchange were made at 8 h solar time (em) and at md. Drought stress was imposed during 20 days. Finally, after the last measurement under drought stressed conditions, plants were rewatered and measurements were performed again after 2 days. In addition, leaves were collected at pd, em, and md for the measurement of sugars concentration, osmolality and RWC during the first and the last days of the drought period (when plants were well watered and just before rewatering, respectively). Leaves for RWC were previously measured with ultrasounds and water potential was also measured at em.

A second complementary experiment was carried out during summer 2015 on a well watered specimen located inside a plant chamber at low CO_2_ concentration (40–80 μmol mol^-1^). Water potential, ultrasounds and sugars concentration were measured on 10 leaves at pd and at md in order to obtain the response of a well watered plant when photosynthetic activity was impaired due to the almost complete absence of CO_2_. Under these conditions, the decrease of water potential at midday (Ψ^md^) with respect to that measured at predawn (Ψ^pd^) would be only affected by concentration of solutes due to the loss of water through transpiration and not by the accumulation of photosynthates. For more details of the plant chamber, see section below. The effect of low CO_2_ concentration on plant photosynthesis was previously checked on the same specimen during the previous day by measuring gas exchange at controlled cuvette CO_2_ concentration (C_a_) between 30 and 90 mol mol^-1^).

### Air-Coupled Ultrasonic Measurements

Two attached mature leaves per plant from three plants were selected and marked at the beginning of the experiment in order to perform all the pd and md ultrasonic measurements systematically on the same leaves. Magnitude and phase of the transmission coefficient were measured in the frequency domain (Sancho-Knapik et al., 2012). Then, in real time, the computer program adjusted theoretical curves to the measured curves of magnitude and phase and gave the value of the resonant frequency associated with the maximum transmitance at the peak curve (*f*, Hz) (Sancho-Knapik et al., 2010, 2012). One ultrasonic measurement was taken between 30 and 60 s. Afterward each value of *f* was divided by the value obtained before dawn at the beginning of the experiment when the plants were well watered (*f*_o_, Hz). The *f/f*_o_ (non-dimensional) associated with the maximum transmittance at the peak curve was then obtained (Sancho-Knapik et al., 2011).

**FIGURE 1:**
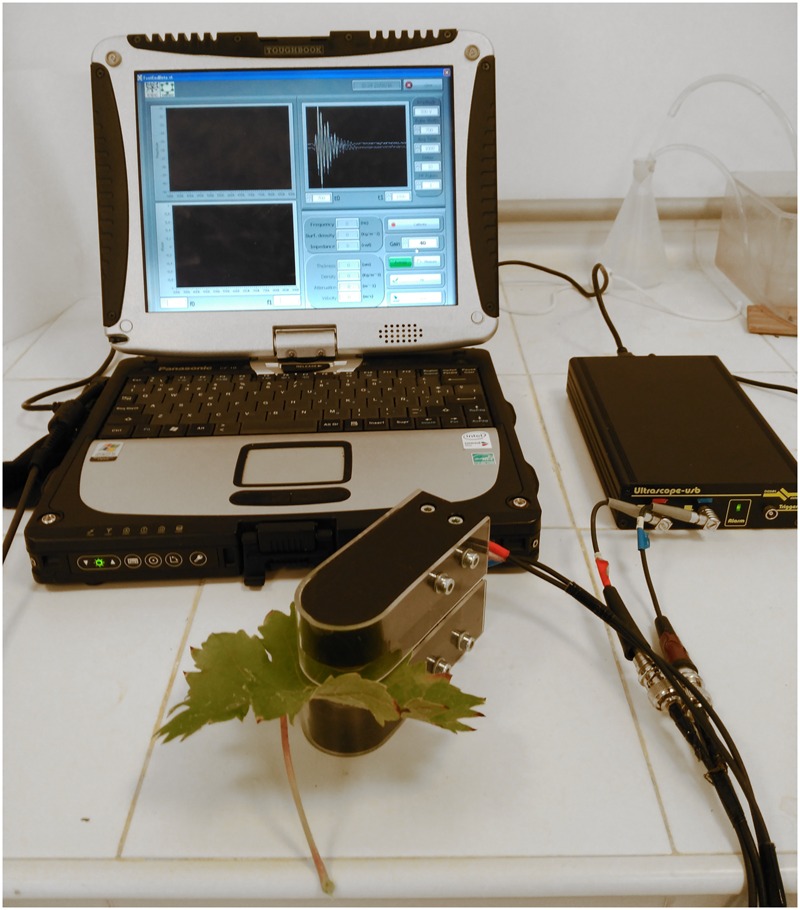
**Schematic picture of the portable air-coupled broadband ultrasonic device**.

The air-coupled ultrasonic system used to obtain the resonant frequency of the leaves in this study was a more portable and easy-to-handle device (Álvarez-Arenas et al., 2016) compared to the one used in previous physiological studies (Sancho-Knapik et al., 2013a,b). The new device (**Figure 1**) consists in one pair of air-coupled transducers embedded in a U-shaped holder which was connected to a small pulser/receiver and to a laptop. The transducers were developed, designed and built at the Spanish National Research Council with a center frequency of 650 kHz, frequency band of 350–950 kHz and area diameter of 20 mm (see Álvarez-Arenas, 2004 for further details). The U-shaped holder maintains the transducers facing each other at a distance of 20 mm providing the necessary robustness that can be easily manipulated without affecting the integrity of the signal. The holder also had a slot in which leaves could be easily positioned between the transducers for the measurements (see Álvarez-Arenas, 2013 and Fariñas et al., 2014 for further details). The pulser/receiver is a commercial device (ULTRASCOPE USB, Dasel Sistemas, Madrid, Spain) which was used to drive the transmitter transducer with a semicycle of square wave (200 V amplitude), tuned to the transducers centre frequency, to amplify (40 dB) the electrical signal provided by the receiver transducer and to digitize this received signal for further processing. It includes pass-band digital (filtering) and extraction of the Fourier transform (using a built-in FFT algorithm). The measurement process was controlled via a graphical user interface (GUI) managed in LABVIEW and also designed by the Spanish National Research Council for this purpose.

### Water Potential, Gas Exchange and Relative Water Content

Predawn and midday leaf water potentials (Ψ^pd^ and Ψ^md^, MPa) were measured in one mature leaf per plant of *V. vinifera* with a Scholander pressure chamber following the methodological procedure described by Turner (1988). Briefly, in this technique a leaf was cut and placed in the pressure chamber with the cut end of the petiole just protruding from the chamber. The pressure inside the chamber was gradually increased by compressed nitrogen gas until the sap just returned to the severed ends of the xylem vessels. In this moment the pressure inside the chamber was recorded and taken as the value of water potential with the opposite sign. Net CO_2_ uptake (*A*, mol CO_2_ m^-2^ s^-1^) and *g*_s_ (mmol H_2_O m^-2^ s^-1^) were measured in two mature leaves per plant located along the middle of the stem with a portable gas exchange system (CIRAS-2, PP-Systems, Herts, UK). Measurements were performed at controlled cuvette CO_2_ concentration (C_a_ = 400 mol mol^-1^), PPFD incident on the leaf surface [∼1300 and 1800 mol photons m^-2^ s^-1^ at em and md, respectively] and ambient relative humidity. RWC was calculated at the beginning and the end of the drought period as a ratio of the difference between leaf fresh weight (FW) minus leaf dry weight (DW) and the difference between leaf turgid weight (TW) minus leaf DW. To obtain RWC, 5 discs per leaf (1.8 cm in diameter) from one half of each blade from two leaves per plant were obtained with a cork borer and subsequently weighed (FW). Afterward discs were introduced in a stove (70°C, 72 h) to obtain DW. The other half of the blade was rehydrated to full turgor by cutting carefully their petioles and submerging them in distilled water. Leaves were wrapped in plastic seal and stored in the fridge overnight. TW was then obtained from 5 disks per leaf.

### Pressure-Volume Curves

Pressure-Volume (P-V) curves were determined using a Scholander pressure chamber following the free-transpiration method described in previous studies (Turner, 1988; Dreyer et al., 1990). The weight and water potential were measured at different levels of RWC, starting at full turgor (TW) and until close to -2.5 MPa were reached. Two leaves per plant were collected at pd from well watered plants. Their petioles were carefully cut and submerged in distilled water until full rehydration. Afterward, leaves were wrapped in plastic seal and left overnight in the refrigerator. After the measurements, leaf DW was obtained by keeping the plant material in a stove (70°C, 72 h). RWC was then calculated as explained above being FW the sample fresh weight at any moment. Additionally, ultrasounds measurements were simultaneously performed in order to relate the values of turgor pressure (*P*) derived from P-V curves with *f*/*f*_o_.

### Leaf Sugars Analysis and Osmolality

Two leaves per plant of three different plants were collected at pd, at em and at md from well watered plants (at the beginning of the experiment) and from drought stressed plants (just before rewatering). Leaves were cut from the plants and were introduced in glass tubes closed hermetically and kept in ice for leaf preservation. Immediately after, tubes were carried to the laboratory and were stored in a freezer at -80°C until analysis. To obtain the expressed leaf sap, frozen leaves were easily broken with a glass rod and they were centrifuged at 5000 rpm for 20 min (Yu et al., 1999). For sugars analysis, 20 μL of the expressed sap extract was filtered by 0.45 μm nylon filters and injected in a HPLC 360 (Kontron, Milan, Italy), equipped with a double piston pump, self-sampler, and regulated furnace at 40°C. The column used was a 250 mm × 4.6 mm i.d., 5 mm, Kromasil Amine. The mobile phase was an isocratic elution of acetonitrile:water (75/25). Detection of sugars (fructose, glucose, and sucrose) was based on a liquid chromatographic method (AOAC, 1990) using a differential refractive index (RI) detector model ERC-7512 (Erma Inc., Tokyo, Japan) at 40°C temperature. The linear range for the concentration determination of each compound was 0.05–2 g/100 ml. Identification of chromatographic peaks was based on retention times by comparison with known standards (Merck, Darmstadt, Germany). An external calibration curve was prepared for each carbohydrate standard to calculate the amount of these compounds present in the leaf extract. The percentage weight:volume (% w:v) of sugars (fructose, glucose and sucrose) was finally obtained. On the other hand, another aliquot (10 μl) was used for measuring the osmolality (mmol Kg^-1^) of expressed sap of em and md samples with a calibrated vapor pressure osmometer (Wescor Vapro model 5500, Wescor, Logan, UT). Osmotic potential of the expressed sap was then calculated from the osmolality using the Van’t Hoff equation (Callister et al., 2006).

### Plant Chamber

The chamber consisted of a Teflon FEP (Fluorinated Ethylene Propylene copolymer) film rolled and stapled into a wood rectangular frame (80 cm × 80 cm × 150 cm). A tape was used to seal the chamber and a 10-cm^2^ hole was left in one of the upper corners to have an open flow. Teflon FEP was used because of its low permeability to liquids and gasses and its excellent transmission in the infrared and visible range of the solar spectrum (Liu et al., 2000). The plant chamber was screwed on a wood base (100 cm × 100 cm) where a few holes were cut to accommodate the plant stem (3.8 cm in diameter) and the air inlet tubes (10 mm in diameter). The base was then cut in half through the stem opening so that it could be moved on and off the plant. Two chest-latches held the base together during operation. A sleeve of sealed-cell foam was placed around the plant stem. The foam was slightly larger in diameter than the opening for the vine stem, enabling the chamber base to compress the sleeve and form a tight seal around the stem. This design separated the plant from the potting soil to eliminate the effects of soil and root respiration on CO_2_ determinations (Miller et al., 1996). Finally, the joining between the chamber and the base was sealed with a plastic adhesive tape.

The air-supply system consisted of an air compressor (model LX 108, Atlas Copco AB, Stockholm, Sweden) with an output of c.a. 2000 l/min and a maximum pressure of 10 bar. This compressor was attached to a “T” connector through an 8-mm-i.d., polyamide tube. From the connector, two polyamide tubes ended in two clear acrylic pipes (38-mm-i.d., and 1.30 m in length) that were filled up with soda lime and molecular sieve (90:10) in order to lower the air CO_2_ concentration inside the chamber. Two more polyamide tubes connected the end of the pipes to the base of the plant chamber. CO_2_ concentration was measured during the experiment with a Carbon Dioxide Probe (GMP343, Vaisala CARBOCAP, Helsinki, Finland) obtaining concentrations levels inside the chamber between 40 and 80 ppm. Two small electric fans (12 cm × 12 cm × 3.8 cm) were located on the wood base to stir the air throughout the chamber. Finally, a small rocking piston (model ROA-P201-BN; Gast Manufacturing Inc., Benton Harbor, MI, USA) with an output of 35 l/min and a maximum pressure of 6.9 bar was used to recirculate the chamber air through another acrylic pipe filled with silica gel in order to dry the chamber air and avoid a water-saturated atmosphere.

### Statistical Analysis

Relationships between mean values of *f/f*_o_ and water potential obtained at predawn (f/$f_{o}^{pd}$, Ψ^pd^) and at md (Ψ^md^, f/$f_{o}^{md}$) were adjusted to a linear function. One-way ANOVAs were performed to compare Ψ, *f/f*_o_, *A, g*_s_, RWC, π and sugars concentration among the different times of the day (pd, em, and md) for plants well watered and drought stressed plants just before rewatering. Multiple comparisons were carried out among the different times of the day using the *post hoc* Tukey’s Honestly Significant Difference test. A Student’s *t*-test was used to compare the same parameters between well watered and drought stressed plants. All statistical analyses were performed with the program SAS version 8.0 (SAS, Cary, NC, USA).

## Results

**FIGURE 2:**
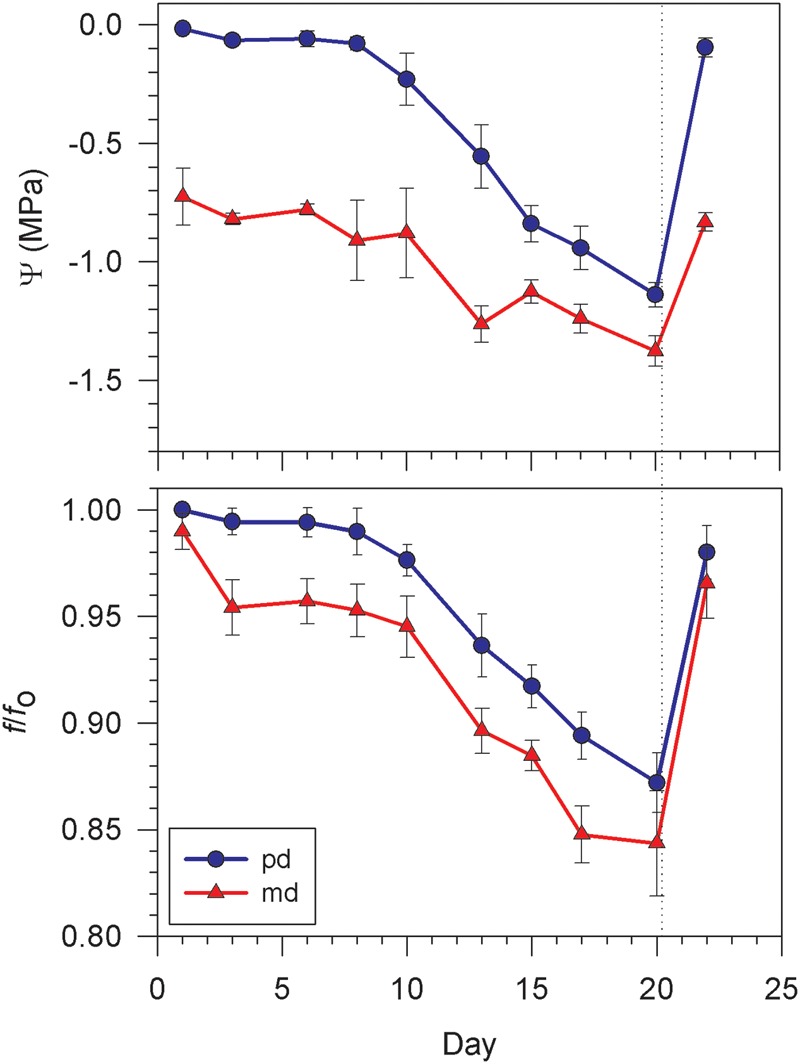
**Evolution of water potential (Ψ) and standardized frequency (*f*/*f*_o_) to drought for *Vitis vinifera* cv. Grenache measured at pd (blue circle) and md (red triangle) along the drought stress experiment.** Values are expressed as mean ± standard error of six measurements for *f*/*f*_o_ and three for Ψ. Dotted line indicates plant rewatering.

**FIGURE 3:**
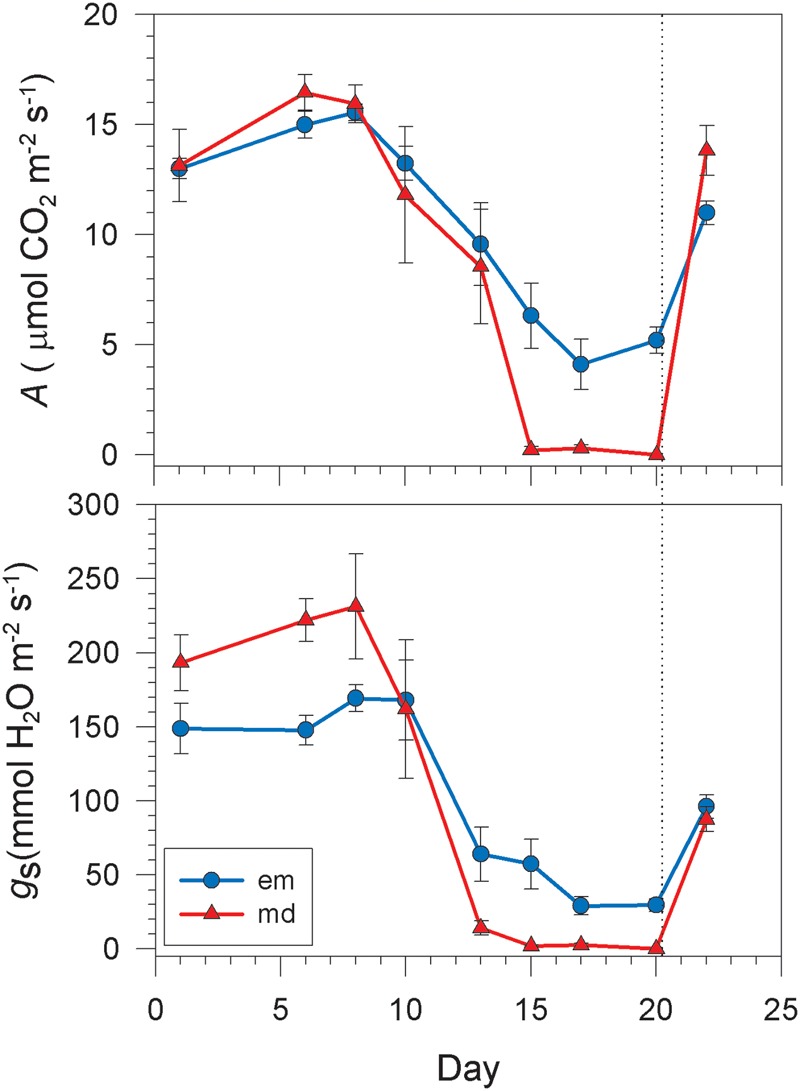
**Evolution of Net CO_2_ uptake (*A*) and stomatal conductance (*g*_s_) to drought for *Vitis vinifera* cv. Grenache measured at early morning (blue circle) and md (red triangle) along the drought stress experiment.** Values are expressed as mean ± standard error of six measurements.

The evolution of pd water potential (Ψ^pd^) and *f/f*_o_ (*f*/$f_{o}^{pd}$) for *Vitis vinifera* cv. Grenache showed that plants started the drought period with values close to full turgor (Ψ^pd^ = -0.01 ± 0.00 MPa, f/$f_{o}^{pd}$ = 1.00 ± 0.00) (**Figure 2**). Six days after the last watering, Ψ^pd^ and f/$f_{o}^{pd}$ became slightly lower, reaching values of -0.06 ± 0.03 MPa and 0.994 ± 0.007 respectively. From here, Ψ^pd^ and f/$f_{o}^{pd}$ dropped to -1.14 ± 0.05 MPa and 0.872 ± 0.014, respectively, at the end of the drought period. Two days after rewatering, values of Ψ^pd^ and f/$f_{o}^{pd}$ became similar to those obtained at the beginning of the experiment. Midday measurements for water potential and ultrasounds showed a similar trend than that found at pd (**Figure 2**). Thus, plants experienced a slight decrease of Ψ^md^ and f/$f_{o}^{md}$ during the first days of the experiment followed by a drop at the end of the dry period and a recovery to the initial values after rewatering. Regarding gas exchange measurements, plants started the drought period with values of *A* and *g*_s_ at em of 13.0 ± 0.5 mol CO_2_ m^-2^ s^-1^ and 148.8 ± 17.1 mmol H_2_O m^-2^ s^-1^, respectively (**Figure 3**). The values of *A* and *g*_s_ measured at md at the beginning of the experiment were very similar than those measured at em (**Figure 3**). These values remained practically constant at em and md during the first 6 days of the experiment. Since this day, both *A* and *g*_s_ started to decline, especially at md, until they reached values close to zero at md (**Figure 3**). A recovery was observed at em and md two days after rewatering, with values of *A* and *g*_s_ at em of 10.98 ± 0.54 mol CO_2_ m^-2^ s^-1^ and 96.2 ± 8.1 mmol H_2_O m^-2^ s^-1^, respectively (**Figure 3**).

**FIGURE 4:**
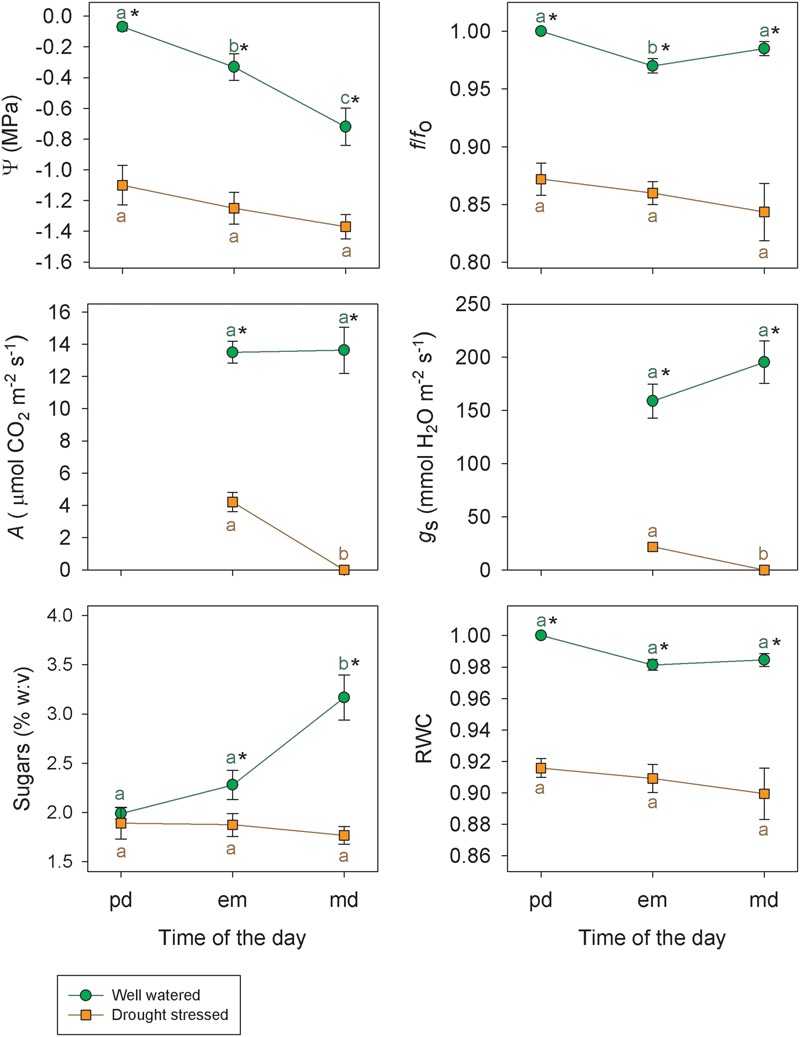
**Water potential (Ψ), standardized frequency (*f*/*f*_o_), Net CO_2_ uptake (*A*), stomatal conductance (*g*_s_), percentage weight:volume (% w:v) of leaf sap sugars (fructose, glucose and sucrose) and relative water content (RWC) for well watered (green circle) and drought stressed (orange square) plants of *Vitis vinifera* cv. Grenache during three moments of the day: pd, em, and md.** Measurements on well watered and drought stressed plants were taken the first and the last day of the drought period, respectively. Values are expressed as mean ± standard error. Asterisks indicate statistically significant differences between well watered and drought stressed plants (Student’s *t*-test, *p* < 0.05). Different letters indicate significant differences among pd, em, and md for well watered and drought stressed plants (Tukey test, *p* < 0.05).

Plants well watered – at the beginning of the drought period – did not show statistically significant differences at *p* < 0.05 between pd and md for *f/f*_o_ and RWC (**Figure 4**). However, significative variations between pd and md (*p* < 0.05) were found for Ψ (from -0.07 ± 0.02 to -0.84 ± 0.12 MPa) and sugars (from 1.99 ± 0.06 to 3.17 ± 0.23 in % w:v) (**Figure 4**). Differences in π obtained from the osmolality of the expressed sap were also found between em and md (*p* < 0.05) (**Table 1**). On the other hand, at the end of the drought period plants did not show statistically significant differences at *p* < 0.05 between pd, em, and md for *f/f*_o_, RWC, Ψ, sugars (**Figure 4**) and between em and md for π (**Table 1**). In other words, although *f/f*_o_ and RWC did not show changes between pd and md neither for well watered nor for drought stressed plants, plants well watered showed a great decrease in Ψ and a great increase in sugars between pd and md when compared to drought stressed plants (**Figure 4**).

**Table 1:** Osmotic potential (π, MPa) obtained from the osmolality of the expressed leaf sap measured on well watered and drought stressed plants of *Vitis vinifera* cv. Grenache during two moments of the day: em and md.

	em	md
π (Well watered)	-1.17 ± 0.05 a	-1.33 ± 0.06 b^∗^
π (Drought stressed)	-1.19 ± 0.05 a	-1.18 ± 0.05 a

*Measurements on well watered and drought stressed plants were taken the first and the last day of the drought period, respectively. Values are expressed as mean ± standard error. Asterisks indicate statistically significant differences between well watered and drought stressed plants (Student’s *t*-test, *p* < 0.05). Different letters indicate significant differences among em and md for well watered and drought stressed plants (Tukey test, *p* < 0.05).*

**FIGURE 5:**
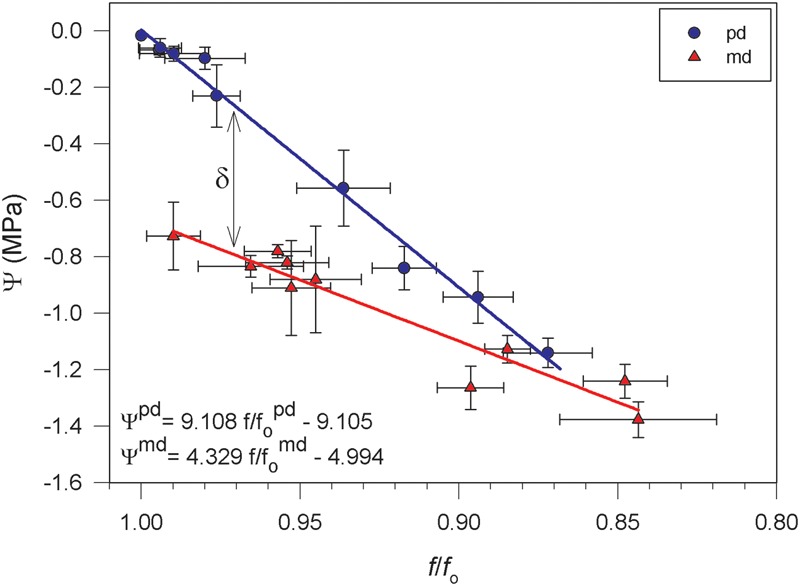
**Relationships between water potential (Ψ) and standardized frequency (f/f_o_) for *Vitis vinifera* cv. Grenache measured at pd (blue circle) (*R*^2^ = 0.99, *p* < 0.001) and md (red triangle) (*R*^2^ = 0.91, *p* < 0.001).** δ indicates the difference between the value of md Ψ and pd Ψ for a given *f*/*f*_o_. Values are expressed as mean ± standard error.

**FIGURE 6:**
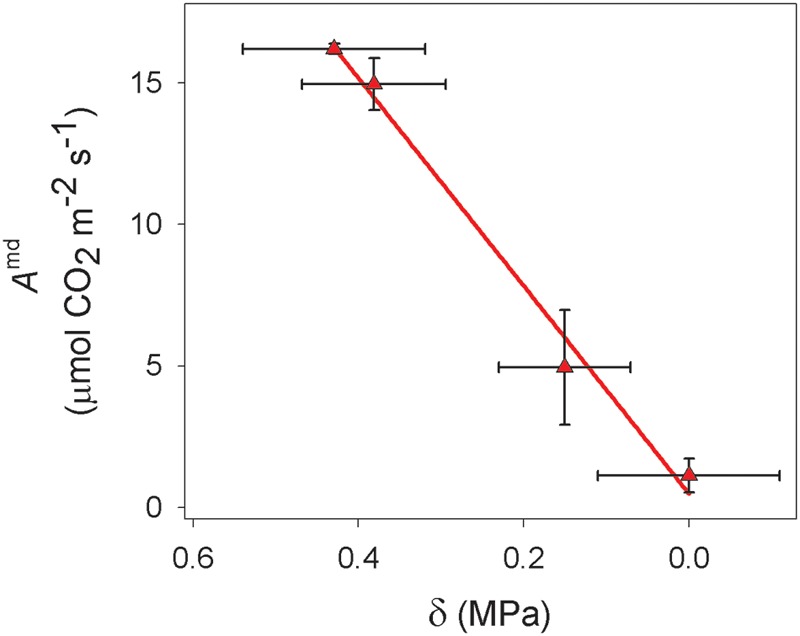
**Relationship of the difference between the value of md and pd water potential for a given standardized frequency (δ) and Net CO_2_ uptake measured at midday (*A*^md^) for *Vitis vinifera* cv. Grenache.** Values are expressed as mean ± standard error.

Two linear relationships between *f/f*_o_ and Ψ at pd and md were obtained with the values of both parameters measured during the drought cycle (**Figure 5**; *R*^2^ = 0.99, *p* < 0.001 for pd and *R*^2^ = 0.91, *p* < 0.001 for md). It was observed that a decrease in *f/f*_o_ was associated with a decrease in Ψ and a convergence between the relationships at pd and at md toward lower values of *f/f*_o_. In addition, **Figure 5** shows that for a given value of *f/f*_o_ there was a corresponding value of Ψ at pd and a different value at md. The difference between the two values of water potential for a given *f/f*_o_ (δ was related to the value of net CO_2_ uptake measured at midday (*A*^md^) (**Figure 6**). This relationship showed that the highest value of δ (0.4 ± 0.1 MPa) occurred at the time of the highest value of *A*^md^ (16.2 ± 0.2 mol CO_2_ m^-2^ s^-1^). When *A*^md^ became lower due to drought stress, δ also decreased, reaching values of zero when *A*^md^ also reached zero.

Finally, the grapevine plant forced to live for 1 day under a CO_2_ concentration (between 40 and 80 mol mol^-1^) lower than the atmospheric one (*p* < 0.05) showed statistically significant differences at *p* < 0.05 between pd and md for Ψ (from -0.11 ± 0.01 to -0.56 ± 0.05 MPa) and *f/f*_o_ (from 0.990 ± 0.002 to 0.939 ± 0.006) (**Table 2**). However, the concentration of leaf sap sugars under low CO_2_ concentration did not show statistically significant differences between pd and md (**Table 2**). The measurement of gas exchange at md under low CO_2_ concentrations during the previous day confirmed the absence of net CO_2_ uptake (-0.2 ± 0.6 mol CO_2_ m^-2^ s^-1^).

**Table 2:** Leaf water potential (Ψ), standardized frequency (*f/f*_o_), net CO_2_ uptake (*A*) and percentage weight:volume (% w:v) of leaf sap sugars measured on well watered plants of *Vitis vinifera* cv. Grenache at low CO_2_ concentration (*C*_a_ = 40–80 μmol mol^-1^).

	pd	md
Ψ (MPa)	-0.11 ± 0.01	-0.56 ± 0.05
*f/f*_o_	0.990 ± 0.002	0.939 ± 0.006
*A* (μmol CO_2_ m^-2^ s^-1^)	0	-0.2 ± 0.6
Sugars (% w:v)	5.31 ± 0.25	4.98 ± 0.19

*Measurements were performed at pd and md. Values are expressed as mean ± standard error (*n* = 10). For sugar analysis, not significant differences (*P* < 0.05) were found between pd and md.*

## Discussion

The air-coupled broad-band ultrasonic spectroscopy has proved useful in this study to monitor how changes in the *f/f*_o_ varied with water potential (Ψ) in attached transpiring leaves of *Vitis vinifera*. When plants became drought stressed, Ψ and *f/f*_o_ varied simultaneously (**Figure 2**) resulting in good correlations between the two variables (**Figure 5**), as shown for detached leaves during previous dehydration studies (Sancho-Knapik et al., 2010, 2013a). The development of a new smaller and easy-to-use ultrasonic device used in this work provided the possibility of measuring attached leaves under field conditions, in contrast to previous studies made under laboratory conditions with a more complex and non-portable apparatus (Sancho-Knapik et al., 2010, 2011, 2013a,b). It should be noted that, at present, this device cannot be used for plant species with small leaves due to the size of the transducers. This is a matter that deserves further technical improvements.

**FIGURE 7:**
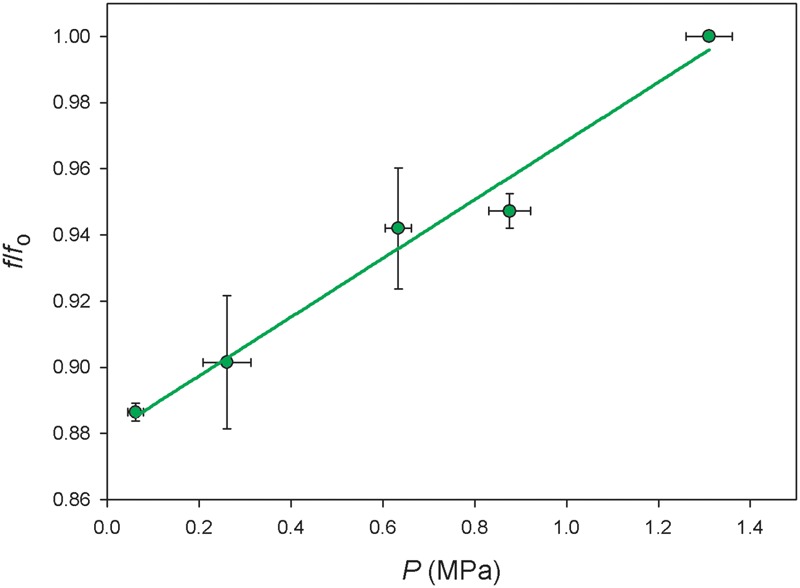
**Relationship between turgor pressure (*P*) and standardized frequency (*f*/*f*_o_) measured on six leaves of *Vitis vinifera* cv. Grenache (*R*^2^ = 0.98, *p* < 0.01).** Values are expressed as mean ± standard error.

The methodology used in this study is based in two main assumptions which were validated by the results obtained. On the one hand, the ultrasonic technique was only measuring one of the components of Ψ the turgor pressure (*P*), and on the other hand, *Vitis vinifera* plants developed diurnal osmotic adjustments that facilitated the maintenance of leaf turgor during md. Both assumptions have emerged from the occurrence of two different Ψ values, one at pd and other at md, for a given value of *f/f*_o_ (**Figure 5**). Regarding the first assumption, the positive linear relationship found between *P* and *f/f*_o_ (**Figure 7**) showed that a higher *f/f*_o_ value can be related to an increased *P*-value, independently of the time of the day when measurements were carried out (i.e., pd or md). Taking this into account, plants with low values of water potential at md (Ψ^md^) but with relatively higher values of f/$f_{o}^{md}$, would have *P*-values close to full turgor. Therefore, the difference between both Ψ values (δ) could not be associated with changes in *P* because *f/f*_o_ remained constant.

Concerning the second assumption, we found that δ was dependent on net CO_2_ uptake (*A)* (**Figure 6**), which could be used as a rough estimator of photosynthetic activity. To the extent of out knowledge, this is indeed the first time in which a quantitative relationship between δ and *A* has been described, and this relation might change for other cultivars (e.g., Syrah, data not shown) or species with different photosynthetic rates. Considering (i) that *P* was not the factor leading to the occurrence of δ in **Figure 5**, and (ii) the actual existence of a relationship between δ and *A*, we propose that the remaining component of Ψ, i.e., the osmotic potential (π), may have induced the decrease of Ψ^md^ with respect to Ψ^pd^ through the synthesis of carbohydrates. Indeed, we have found a significant decrease in π between em and md estimated with the osmometer (**Table 1**), despite the discrepancy between this and other methods for the measurement of π (Callister et al., 2006). These dynamic changes in π can be explained through the daily accumulation of solutes due to photosynthesis in order to maintain *P* (i.e., the so-called “diurnal osmotic adjustment” according to Acevedo et al., 1979). The solutes that can lower π are divided in four classes (Sanders and Arndt, 2012): (i) sugars, that are considered the main component for the change in diurnal osmotic adjustment (Acevedo et al., 1979); (ii) organic acids, which could represent less than -0.01 MPa according to Acevedo et al. (1979); (iii) inorganic ions which play a minor influence in osmotic adjustment (Warren et al., 2011) and (iv) free amino acids (specially proline) which mainly increase at higher drought stress levels in prolonged dry periods (Irigoyen et al., 1992; Aranjuelo et al., 2011; Masoudi-Sadaghiani et al., 2011). In this study, the high photosynthetic activity measured in fully irrigated plants induced the production and accumulation of sugars at md (**Figure 4**). These solutes may have induced a decrease in π between pd and md (the diurnal osmotic adjustment), which was noticeable due to the decrease of the water potential at midday (Ψ^md^) with respect to that measured at predawn (Ψ^pd^) without any significant reduction in *f/f*_o_ and RWC (**Figure 4**). That is, water loss by transpiration did not induce a significant reduction in Ψ^md^. Furthermore, when RWC at md in well watered plants (**Figure 4**) is expressed in terms of the value of Ψ given by the P-V curves (data not shown), this value (-0.30 ± 0.06 MPa) is less negative than the one measured during the experiment (-0.72 ± 0.12 MPa, **Figure 4**). The difference between both values may then represent the diurnal osmotic adjustment due to the effect of solute accumulation due to photosynthesis. Moreover, this difference is similar to the one estimated with sugar concentrations for well watered plants (**Figure 4**) using the equation of Acevedo et al. (1979), which yielded a theoretical change of ca. 0.3 MPa in the osmotic potential between pd and md. The adjustment observed was larger at the beginning of the experiment, when plants had higher values of net CO_2_ uptake (*A*) and increased sugar concentrations (**Figures 3** and **4**, respectively) than at the end of the drought period when net CO_2_ assimilation was almost negligible (**Figure 3**). Consequently, in drought stressed plants it was not detected neither an accumulation of sugars in leaves (**Figure 4**) nor an increment in π (**Table 1**) and, therefore, Ψ was not lowered between pd and md. In this situation, the expected values of Ψ based on the measurements of f/$f_{o}^{md}$ corresponded with the measured Ψ^md^ values.

The measurements obtained from the experiment of a well-watered Grenache plant under low CO_2_ concentration inside a chamber confirmed that changes in *f/f*_o_ were associated to changes in *P*. The low CO_2_ concentration inside the plant chamber induced close-to-zero net CO_2_ uptake. Despite pd values of sugar content higher than those seen in the previous experiment, here an accumulation of sugars at md was not seen (**Table 2**). Acevedo et al. (1979) also reported a lack of accumulation in sugars and a lack of π diurnal change when a close-to-zero net CO_2_ uptake was induced in sorghum leaves by artificial plant shading, suggesting an association between photosynthesis and the observed diurnal osmotic adjustment. Therefore, it is then logical to assume that in *V. vinifera* under low CO_2_ concentration no decrease occurred in π between pd and md, leaving *P* as the only responsible component of the decrease in Ψ. Observed changes in Ψ^md^ were indeed very small and compared to those seen in water-stressed plants. Under these circumstances, measured values of f/$f_{o}^{md}$ should reflect measured values in Ψ^md^ without the confounding factor of diurnal osmotic adjustment. Consistent with this idea, if *f/f*_o_ and Ψ values of the Grenache specimen at low CO_2_ concentration measured at md (**Table 2**) are plotted over the relationships between *f/f*_o_ and Ψ for that appears in **Figure 5**, it can be observed that values at md from **Table 2** fitted the relationship obtained at pd.

It should be noted that, although the seasonal osmotic adjustment may be a common phenomenon in many plant species (Hsiao et al., 1976) including *V. vinifera* (Martorell et al., 2015), the diurnal osmotic adjustment has been studied and detected only in a few species during the last decades (Acevedo et al., 1979; Rada et al., 1985; Koroleva et al., 2002). This physiological response has been interpreted as an adaptive mechanism for plants that may experience drought stress by means of an active increase in the concentration of cell solutes that may maintain positive turgor potentials above thresholds for stomatal closure and growth cessation (Rada et al., 1985). In contrast with these previous studies that detected the osmotic adjustment through the direct measurement of π in the laboratory, our investigation has served to suggest this phenomenon on attached transpiring leaves under field conditions.

## Conclusion

This study demonstrates for the first time that plant water status of *V. vinifera* can be monitored on transpiring leaves under field conditions through the measurement of ultrasonic frequencies. Moreover, our results suggest the existence of a diurnal osmotic adjustment in this species associated to sugars production, which plays a key role in the decrease found in Ψ between pd and md in well-watered plants. These facts, together with the possibility of measuring ultrasonic frequencies continuously (Fariñas et al., 2014) make the ultrasonic technique a promising tool to be used in plant sciences.

## Author Contributions

DS-K, HM, JP-P, and EG-P conceived the study and participated in its design. MF and TA-A developed and applied the ultrasonic system. All authors analyzed and interpreted the data. DS-K drafted the manuscript and JP-P, MM, TA-A and EG-P critically revised the manuscript. All authors read and approved the final version of the manuscript.

## Conflict of Interest Statement

The authors declare that the research was conducted in the absence of any commercial or financial relationships that could be construed as a potential conflict of interest.

## Abbreviations

*A*: net CO_2_ uptake
*C*_a_: ambient CO_2_ concentration
em: early morning
*f/f*_o_: standardized frequency
*g*_s_: stomatal conductance
md: midday
pd: predawn
*P*: turgor pressure
RWC: relative water content
π: osmotic potential
Ψ: water potential
